# Energy Balance of Canadian Armed Forces Personnel during an Arctic-Like Field Training Exercise

**DOI:** 10.3390/nu12061638

**Published:** 2020-06-02

**Authors:** Mavra Ahmed, Iva Mandic, Elliot Desilets, Ingrid Smith, Wendy Sullivan-Kwantes, Peter J. Jones, Len Goodman, Ira Jacobs, Mary L’Abbé

**Affiliations:** 1 Department of Nutritional Sciences, University of Toronto, Toronto, ON M5S 3E2, Canada; mavz.ahmed@mail.utoronto.ca; 2 Faculty of Kinesiology and Physical Education, University of Toronto, Toronto, ON M5S 2W6, Canada; iva.mandic@mail.utoronto.ca (I.M.); ira.jacobs@utoronto.ca (I.J.); 3 Department of Human Nutritional Sciences, University of Manitoba, Winnipeg, MB R3T 6C5, Canada; umdesil6@myumanitoba.ca (E.D.); peterjones@nfh.ca (P.J.J.); 4 Defence Research and Development Canada, Toronto Research Centre, Toronto, ON M3K 2C9, Canada; ingrid.smith@drdc-rddc.gc.ca (I.S.); wendy.sullivan-kwantes@drdc-rddc.gc.ca (W.S.-K.); len.goodman@drdc-rddc.gc.ca (L.G.); 5 Department of Pharmacology and Toxicology, Faculty of Medicine, University of Toronto, Toronto, ON M5S 3E2, Canada; 6 Department of Nutritional Sciences, Faculty of Medicine, University of Toronto, Medical Science Building, 1 King’s College Circle, Room 5368, Toronto, ON M5S 1A8, Canada

**Keywords:** energy balance, energy intake, energy expenditure, dietary intake assessment, nutrition, military training, cold environment, measured food intake and waste

## Abstract

Operating in temperature extremes frequently leads to a discrepancy in energy balance. Investigating the effects of operating in extreme cold temperatures on metabolic requirements has not been well described in Canadian Armed Forces (CAF) personnel. The objective was to accurately assess energy deficits using the “gold standard” methodology for measuring energy intake (EI) and energy expenditure (EE). Nutritional intake of a convenience sample of 10 CAF Class A Reservists, completing a basic military qualification (land) course under winter weather conditions, was assessed using the daily measured food intake/food waste collections. EE was measured by the doubly-labelled water method. Average EI was 2377 ± 1144 kcal/day, which was below the EE (4917 ± 693 kcal/day), despite having ~5685 kcal available in the field rations. A significant body weight loss of 2.7% was associated with the average daily energy deficit of 2539 ± 1396 kcal. As a result, participants demonstrated voluntary anorexia. Such results may have important implications for the impairment of performance and health under longer duration operations.

## 1. Introduction

During field training or operations, energy expenditures can range from less than 6000 to ~10,000 kilocalories (kcal) per day in different environmental conditions because of extended periods of physically demanding activities with limited sleep or rest [1,2,3]. Moreover, these increases in energy expenditure are aggravated by extreme environmental temperatures [1,4,5]. For example, energy requirements in the cold environment (temperature <10 °C, including arctic conditions) have been reported to range from ~4000 to >6000 kcal/day [6,7,8,9,10,11], but can significantly increase due to the increased metabolic rate associated with functioning in heavier clothing/boots, additional demands due to physical activity on snow/ice-covered terrain and additional heat production to regulate body temperature [12,13,14]. 

Pre-packaged field rations are frequently provided to Canadian Armed Forces (CAF) infantry personnel during military missions or field training exercises but are not to be used exclusively beyond 30 consecutive days [15,16]. However, the provision of fresh foods is not always possible [6] and missions may be longer than intended [6]. In addition to the standard food allowances, incremental allowances may be provided under conditions when it is suspected that standard allowances may be insufficient to fuel the energy demands of metabolically challenging operations under environmental extremes [6,16,17].

It has been repeatedly reported that military personnel operating in temperature extremes frequently experience a discrepancy between the amount of food that is available versus the amount that is consumed [2]. Factors previously suggested as mediating this phenomenon include the palatability/variety of rations provided, cooking method/time required for food preparation, and load carriage [6,15,18]. The nutrient intakes of CAF personnel may be further compromised due to field rations partially eaten, or portions discarded during field operations or training [17,19]. These factors have repeatedly been reported in the literature to play a role in contributing to voluntary anorexia (defined as the failure to consume foods that are offered or readily available under situations of extreme stress [3]), which can have negative psychological and physiological consequences, thereby impacting operational readiness and performance [2,3,20]. 

Investigating the effects of extreme cold on operational requirements is challenging in real-life settings and thus has not been well described in CAF personnel. The majority of the studies conducted with Canadian military personnel in extreme cold environments did not accurately assess energy and nutrient intakes coupled with accurate measures of energy expenditure in the same study [7,15]. Therefore, it is difficult to assess the extent to which energy deficits observed during field operations can be attributed to discrepancies in energy intake/expenditure or to imprecision in measurement.

Using the current gold standard in dietary assessment methodology [21], the multi-day measured food intake/waste collection method, the primary objectives of this study were to: (1) accurately assess energy and nutrient intakes, of a convenience sample of CAF personnel during completion of a 5-day basic military qualification course (land) under winter weather conditions, coupled with accurate assessments of energy expenditure using doubly-labelled water and; (2) assess the amount of energy and nutrient from food and beverage items that were discarded (i.e., ration components discarded prior to or during the training phase) from rations.

## 2. Methods

### 2.1. Study Participants

A convenience sample (i.e., those who were available to participate) of 20 CAF participants from Class A Reservists initially volunteered to participate in this study. Of the 20, 18 completed the exercise and two dropped out due to medical reasons. Data are presented for 10 participants with complete energy expenditure measurements. The volunteers were not selected to be representative of the entire CAF regular force population but were a convenience sample of Class A Reservists. This research was conducted during a regularly scheduled CAF basic military qualification training course at Canadian Forces Base (CFB) Meaford between 26 and 30 January 2015.

Participants remained in the field for the duration of the study (~0600 on day 3 to ~1800 on day 7). Weather information was collected daily during the duration of the field study which took place from 26 to 30 January 2015. The average mean temperature was −11 °C, with a minimum of −22 °C, a maximum of −2 °C, and a wind-chill of −25.9 °C. All participants provided written informed consent to participate. Research ethics review boards at the University of Toronto (#31260) and Defence Research and Development Canada (DRDC) (#2015-001) approved the study protocol. All procedures performed were in accordance with the ethical standards of the institutional and/or national research committee and with the 1964 Helsinki declaration and its later amendments or comparable ethical standards.

### 2.2. Experimental Protocol

This is an observational cross-sectional study where the pre-study phase (immediately before the training phase) involved the consent process, completion of a Physical Activity Readiness Questionnaire-Plus (PAR-Q+) [22], collection of demographics and anthropometric data, and measurement of body composition (assessed using deuterium isotope dilution). PAR-Q+ was administered prior to enrollment in the study and used as inclusion criteria to determine if the participants were fit for exercise without requiring a secondary medical examination for clearance to participate. 

**Figure 1:**
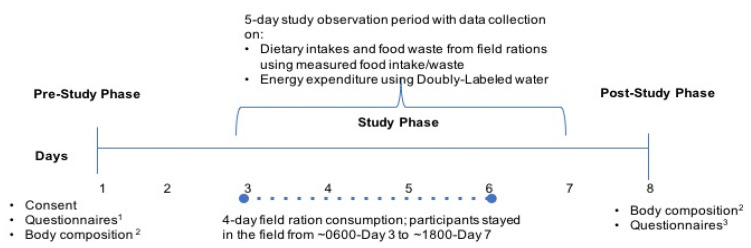
Winter weather (arctic-like) field training exercise experimental protocol. ^1^ Questionnaires on socio-demographics, PAR-Q+—physical activity readiness questionnaire; ^2^ body composition assessed using deuterium isotope dilution procedure; ^3^ end of study questionnaire included questions on body weight maintenance; dietary intakes and food waste from field ration consumption was assessed using a measured food intake/waste collection method; energy expenditure was measured by doubly labelled water (DLW). Study Participants (n = 10) were Class A Reservists, completing a CAF basic military qualification course during a 5-day training exercise.

While the training phase was five days in length, four days of data on dietary intakes were collected and are presented in this manuscript as participants came back to the barracks by ~1800 on Day 5 and would have consumed non-IMP foods for dinner. Data on energy expenditure were collected until the morning of Day 5. Data on dietary intakes were collected using a measured food intake/food waste method and energy expenditure was measured using doubly labelled water (DLW), detailed in the following sections. The post-study phase consisted of measurements of body weight, height and body composition and completion of end of study questionnaires. An overview of the study protocol is shown in Figure 1 and a detailed description of this study can also be found in the technical report prepared by University of Toronto for DRDC [23].

### 2.3. Demographic and Anthropometric Assessments

Participants were asked to complete a standardized demographics questionnaire that included information on age, gender, ethnicity, education and marital status. Anthropometric assessments included measured height, weight, and body fat percentage. Body weight and height were measured before and after the training exercise, without shoes and with participants wearing their standard issued clothing, using standard, calibrated equipment (stadiometer) and weight scales (HealthOMeter Continental Scale Corp., Bridgeview, IL, USA). Body mass index (BMI) was calculated as the body weight (kg) divided by the height (m) squared. 

### 2.4. Dietary Intake Assessment

For the duration of the training exercise, participants selected their choice of individual meal pack (IMP) and/or light meal combat (LMC) rations and had a choice from 6 IMPs per meal type (e.g., 6 breakfast, 6 lunch and 6 dinner) and two LMCs over the training period. The foods and beverages within these rations are of precisely known quantity and nutrient composition. Although, participants were asked to only consume the beverages (e.g., sports drink, coffee, tea, vanilla cappuccino) provided within the rations, they were able to have water *ad libitum*. Due to logistical issues, water intake was not measured. Participants were asked to refrain from the consumption of foods other than those found in rations, as well as dietary supplements and performance enhancers. However, if they did so, participants were asked to provide all wrappers for any foods/beverages they consumed outside of their selected IMPs/LMCs. 

The measured food intake/food waste was calculated from the amount unconsumed subtracted from the known quantity of each menu item selected during the field training. Participants discarded the selected rations/IMPs and only took to the field the components of each ration that they thought they would consume. Prior to the field training, participants were provided with plastic bags labelled with their study ID and date and were required to keep all partially consumed/unconsumed food items as well as containers of all consumed food items in these plastic bags, which were collected by the study coordinators at the end of each day. If participants shared food (e.g., chocolate bar), participants were asked to provide half the wrapper of that item, whereas the participant who shared that item would provide the other half of the wrapper. In the scenario of shared items, study coordinators estimated the intake of that particular item as equally distributed between participants. Ration components not consumed prior to or during the training phase were considered discarded (also referred to as preferentially selected). For determining the count of discarded ration items, in the event of partial consumption of the product, an item less than 40% consumed was considered as a discarded count. Each day, all unconsumed and/or partially consumed food and beverage items were weighed and recorded by study coordinators to the nearest gram (g) or milliliter (mL), using a standard food scale (PrepTech, PT-800, Newport Beach, CA, USA). If the container was empty, the item was considered completely consumed. 

Food intake was entered by two trained coders using a nutrient software program (ESHA© Food Processor SQL, version 10.13.1, 2013, ESHA© Research, Salem, OR, USA), which was pre-loaded with nutritional information for all the food and beverage components found within the field rations and double-checked and analyzed by the study investigator (MA). The nutrient values of food and beverages in the combat/field rations were provided by CAF Directorate of Food Services based on manufacturer specifications and/or by chemical analyses and were available as a Nutrition Facts table on the ration components.

### 2.5. Energy Expenditure and Body Composition Assessments

Total daily energy expenditure (TDEE) was measured using the doubly labelled water (DLW) technique (as detailed in previous technical reports [23,24]). Briefly, between 0500 and 0600 h (before the commencement of the training exercise), ten participants (out of 18 participants) provided baseline urine samples and then ingested 2.0 g H_2_O_18_ per estimated kg total body water, and 0.12 g ^2^H per estimated kg total body water. Urine was then collected approximately 24 h post-dose, 72 h post-dose, and 120 h post-dose. Average TDEE was calculated for the 4-day study period (days 3–7). Physical activity level (PAL) was calculated as TDEE/resting metabolic rate (RMR), where RMR was estimated from De Lorenzo’s equation [25]. PAL quantifies physical activity by expressing TDEE as a multiple of RMR. Physical activity energy expenditure was estimated as TDEE–RMR. 

Body composition (including percent body fat) was assessed before and after the training exercise using the deuterium isotope dilution procedure [23]. Fifteen participants provided baseline saliva samples on day 3 and then ingested standard doses of 0.12 g ^2^H per estimated kg total body water. Participants then provided saliva samples 3 and 4 h post-dose. This procedure was repeated at the end of the 5-day study period to compare body composition from the start and the end of the training exercise. 

Urine samples were collected from 5 participants who did not receive any of the isotope treatments to enable the measurement of changes in the background abundance of ^2^H and ^18^O in body water due to isotopic enrichment differences in the local drinking water. These participants served as the control group during the field study.

### 2.6. Statistical Analyses

Energy and nutrient intake data are presented as mean ± standard deviation (SD) or as a percentage of total energy. Macronutrient intakes were assessed in comparison with DRI recommendations for Average Macronutrient Distribution Ranges (AMDR) [26]. The frequencies of discarded ration items were obtained from individual counts of the menu items as returned by each participant at the end of each day. The percentage of ration items discarded was obtained by dividing the count discarded by the total of the item selected. Energy and macronutrients for menu items were multiplied by the frequencies of discarded ration items and their nutritional composition and presented as absolute values. Data were normally distributed. Student’s paired *t*-test was used to assess significant differences in body composition. All data were analyzed using SPSS Statistics (version 24, 2016; IBM Corporation^®^, Armonk, NY, USA). A *p*-value of <0.05 was considered significant. 

## 3. Results

### 3.1. Study Participants

Physical characteristics of the 10 participants with complete energy expenditure data are presented in Table 1. During the 5-day study period, body mass was significantly reduced by 2.7% (−2.1 ± 1.1 kg) and body mass index (BMI) was significantly reduced by 3.0% (−0.8 ± 0.4 kg/m^2^) (*p* < 0.05). There was a 4% decrease in body fat percentage (*p* < 0.05) [23,24]. 

### 3.2. Energy and Nutrient Intakes from Field Rations during Winter Weather (Arctic-Like) Field Exercise

**Figure 2:**
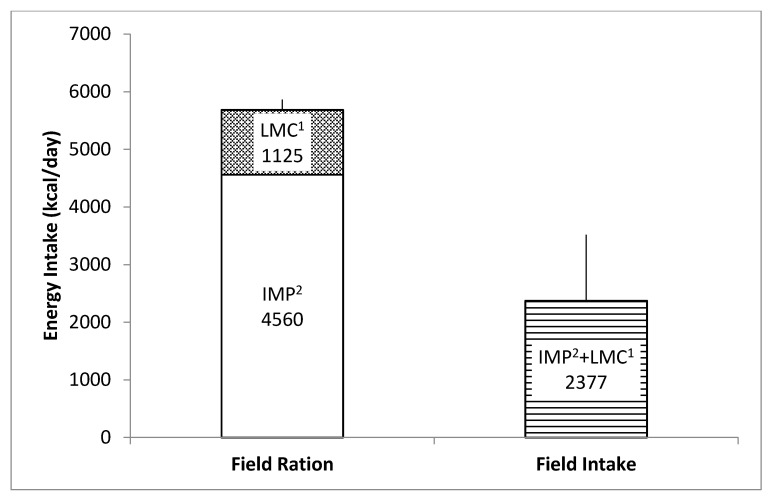
Total energy intake per day available from field rations selected by participants (n = 10) compared to consumed energy intake per day over the four days of dietary intake data collection during the winter weather (arctic-like) training exercise (average temperature −11 °C, with a minimum of −22 °C, a maximum of −2 °C). Field Ration refers to the average energy content available from selected individual meal packs (IMPs)^2^ (4560 kcal) and light meal combat (LMC)^1^ (1125 kcal) rations (calculated using the nutrient values provided by CAF Directorate Food Services), whereas ration intake (2377 kcal) refers to the energy portion consumed by participants (assessed for 4 days using the measured food intake/food waste collection method). Data presented as means ± standard deviation (SD).

Participants were provided with a total of 5685 kcal/day (4560 (IMP) + 1125 (LMC) kcal/day), of which they consumed 42% of the energy available from both IMP + LMC and 52% of the energy available from IMPs only (Figure 2). Average energy intake was 2377 ± 1144 kcal/day (range; 1212–4690 kcal/day), with 52% of total energy from carbohydrates, 32% from fat, and 15% from protein, which were within the AMDR values (Table 2). Total sodium intake was 3542 ± 1722 mg/day, while total sugar intake was 174 ± 79 g/day (Table 3). Intakes of additional nutrients are shown in Table 3. 

### 3.3. Measured Energy Deficits

The mean total daily energy expenditure, as measured using doubly-labelled water, in participants was 4917 ± 693 kcal/day (range; 3761–5985 kcal/day) (for detailed analyses, see Jacobs et al., 2015 and Desilets, E., 2015) [23,24]. The mean energy deficit per participant (n = 10) measured in this study was 2539 ± 1396 kcal/day (Table 4).

## 4. Discussion

The present results indicate, in a convenient sample of CAF Reservists, low energy and nutrient intakes coupled with a high energy expenditure, resulting in a significant loss (2.7%) of total body mass and a 4% decrease in body fat percentage after the 5-day training exercise. These results demonstrate that participants exhibited voluntary anorexia, as has been reported in numerous earlier studies [2,3,9,20,27].

The participants’ loss of total body mass post-study was similar to the findings of previous studies, which have consistently reported energy deficits and weight loss during field operations [8,9,27]. Infantry personnel demonstrated a 1.7% to 2.8% body weight loss during a 10-day field study in Alaska [9,27], comparable to the 2.7% body mass lost in this 5-day study. In contrast, a study by Jones et al. [7], measured a loss of 0.9% body weight in soldiers on a 10-day cold weather military field operation. However, in this latter study, soldiers consumed about 60% of the rations provided, although dietary intake data were collected using self-reported food records, which may suggest potential misreporting compared to the studies conducted in Alaska [27], where soldiers consumed about 40% to 70% of the rations provided and dietary intake data were collected using 24-h recalls confirmed with food waste.

The specific soldier’s job/trade and experience may also play a role in energy intakes because soldiers with trade experience and/or high levels of physical fitness are likely to minimize their energy deficits by ensuring adequate energy intake [11,28]. However, weight losses ranging from 3% to 7% of initial body weight during 14–18 days have been documented in presumably well-trained and experienced US Rangers operating in a variety of environmental temperatures and terrain, although this may be due to food portions being severely limited [29]. Similarly, special operations soldiers were also reported to experience a 1.5% to 5.6% weight loss in 28 days [30]. Longer duration studies have also demonstrated losses of up to 15.6% of body weight during 62 days in experienced soldiers with high levels of physical fitness (US Rangers) [29]. Although we did not assess the physiological or psychological consequences of these reductions in body mass, previous studies have indicated that extreme weight loss during a short-term field exercise can have significant negative impacts on physical performance (e.g., decreases in power output and strength) [1,31,32].

The present study stands out from previous reports because of the use in the same study of accurate and precise methods of quantifying both energy intake (measured food intake/waste collection method with precise control over sharing of foods or inclusion of local/supplemental foods) and expenditure (DLW). Theoretically, in order to obtained the observed weight loss of 2.7% within 5 days (as demonstrated in this study), the participants would have energy deficits of 3150 kcal/day (based on 500 kcal/day to lose 1 lb/week). In this study, participants’ calculated energy deficit was 2540 kcal/day (4917–2377 kcal/day) (measured energy deficit was 2539 kcal/day, based on sample size of 10 participants ingested with DLW), was similar to the theoretical value of 3150 kcal/day.

Participants’ energy intakes were less than 50% of the total energy available for consumption from the selected field rations. The low energy intake (2377 kcal) was similar to that found in other cold-environment experiments, where participants’ energy intakes from field rations ranged from 2009 to 3553 kcal/day [7,15,17,27,33]. The difference of ~100 to 400 kcal (where the energy intake in the present study is lower by 100 to 400 kcal) compared to previous studies may be due to several factors, such as the intake of supplements, local and/or personal foods (which participants were encouraged to refrain from in this study) [7,15], the intensity/duration of the activities (e.g., in the study by Jones et al., the activity level was reported as moderate [7]) and use of self-reported dietary collection methods (e.g., food diaries) that are prone to misreporting [7,15]. There were no direct measurements of aerobic fitness in the current study; however, the participants had a BMI and relative body fat content which was higher than in the other previously cited studies [7,8]. This suggests that they may have voluntarily chosen to exert themselves at a lower absolute metabolic rate than if their fitness was more similar to that of the participants in other relevant studies [34].

Although the average energy content from CAF field rations, if consumed, would have been sufficient to meet the energy requirements of most participants during the training exercise, adequate intake of the available foods did not occur. For example, most of the carbohydrate intake from field rations was obtained as simple sugars; as such, military personnel are potentially missing out on complex carbohydrates from food items such as rice or couscous found within the field rations. For example, vegetable rice and oatmeal were also found to be some of the highest discarded ration items (Supplementary Table S1). Even though these items are highly available for consumption, participants may potentially be missing out on nutrients due to discarding, stripping or partial consumption of menu items that are considered high sources of energy and nutrients (Supplementary Table S1). This study was not designed to assess the reasons behind voluntary under-consumption of field ration packs; however, previous research has pointed to factors such as menu boredom, poor ration palatability, inadequate time to prepare or eat meals, lack of sleep, and operational stress [3,4]. Our previous research demonstrated that CAF soldiers generally report a good acceptability of the variety of menu item options available as well as the palatability of the rations [35]. However, the effect of monotony on field ration acceptability as a result of longer-term consumption of the ration packs needs further consideration.

### Limitations

This study is limited by a small sample size, primarily consisting of CAF Reservists completing a basic military qualification training course and thus, this convenience sample may not be representative of highly-active army personnel. Due to the constraints introduced as a result of field-based research in collaboration with the government and military off-site, we were unable to fully control the experimental protocol (e.g., we were unable to measure water) and subject selection. Considering it was a basic qualification course, several participants may still have been learning the basic elements required to prepare/cook food during field conditions (e.g., having a propane fueled grill), which may also have influenced their energy intake. The participants in this study were asked to refrain from additional foods and/or personal supplements and were solely restricted to the use of field rations for five days in the field. In comparison, soldiers deployed for missions or operations are likely to consume additional personal foods and supplements and/or may even be supplemented with fresh foods depending on the location, condition and availability of incremental allowances, thus the extent to which our results over- or under-estimate energy intake in other CAF troops during harsh environmental conditions is unknown. Although our study demonstrated voluntary anorexia under these conditions, it is limited by our ability to extrapolate to long-term effects during prolonged operations in harsh environmental temperatures on health and performance.

## 5. Conclusions

In the present study, during a winter weather field training exercise, participants demonstrated voluntary anorexia, as their energy intake was significantly lower than that provided in the form of IMPs and LMCs and their energy intake was insufficient in comparison with their energy expenditure, thereby resulting in significant weight loss. Such results have important implications for impairment of performance and health under longer durations than what was examined in this investigation.

Sufficient nutrient intake of CAF military personnel operating in environmental extremes is contingent upon the consumption of the complete ration meal [36]. There may be potential under-consumption of menu items that are significant sources of energy and macronutrients but are discarded. IMPs, if consumed at-least three times a day and in their entirety by military personnel conducting strenuous physical activities under environmental extremes, provide sufficient energy and macronutrients. Enhancing the nutrient density (and possibly taste/palatability) of IMPs with LMC supplementation could help with increasing consumption of specific micronutrients with less food. This research indicates the need for the development and promotion of strategies to increase the food and nutrient intakes of military personnel during training or operational missions in extreme cold to sustain optimal performance.

## Figures and Tables

**Table 1:** Demographic and anthropometric characteristics of winter weather (arctic-like) field training exercise study participants (n = 10) ^1^.

Demographics and Anthropometrics	All (10)	Males (6)	Females (4)
Age (years) ^2^	31 ± 7	29 ± 5	34 ± 9
Height (m) ^2^	1.76 ± 0.08	1.82 ± 0.06	1.68 ± 0.03
Body Mass Pre-Study (kg) ^2^	81.8 ± 9.3	81.9 ± 8.5	81.8 ± 11.7
Body Mass Post-Study (kg) ^2^	79.7 ± 8.8 *	79.7 ± 8.3 *	80.1 ± 11.0 *
BMI Pre-Study (kg/m^2^) ^2,3^	26.6 ± 3.9	24.9 ± 2.4	29.0 ± 4.5
BMI Post-Study (kg/m^2^) ^2,3^	25.8 ± 3.8 *	24.2 ± 2.3 *	28.3 ± 4.4 *
Body Fat (%) Pre-Study	30.1 ± 7.0	29.2 ± 7.9	31.4 ± 6.1
Body Fat (%) Post-Study	26.0 ± 8.0 *	24.9 ± 8.5 *	27.4 ± 8.2 *
Fat Mass Pre-Study (kg)	22.7 ± 7.0	19.3 ± 5.3	27.9 ± 6.4
Fat Mass Post-Study (kg)	19.5 ± 7.7 *	15.9 ± 5.7 *	24.9 ± 7.9 *
Fat Free Mass Pre-Study (kg)	59.1 ± 7.5	62.6 ± 7.0	53.9 ± 5.2
Fat Free Mass Post-Study (kg)	60.3 ± 6.3 *	63.7 ± 5.6 *	55.3 ± 3.2 *

^1^ Study participants (n = 10) were Class A Reservists, completing a CAF basic military qualification course during a 5-day winter weather (arctic-like) training exercise (average temperature −11 °C, with a minimum of −22 °C, a maximum of −2 °C). ^2^ Mean ± standard deviation (SD), ^3^ The BMI should be considered as slightly inflated since body weight was measured while clothed. * Significantly different from pre-study, *p* < 0.05.

**Table 2:** Energy and macronutrient composition of field rations selected and consumed by study participants (n = 10) ^1^.

Field Rations	Energy (kcal)	Carbohydrates (g)	Total Fat (g)	Protein (g)
**Breakfast**	1435 ± 188	221 ± 27	45 ± 13	49 ± 4
**Lunch**	1587 ± 131	227 ± 26	50 ± 9	61 ± 5
**Dinner**	1538 ± 201	234 ± 32	44 ± 10	55 ± 10
**IMP ^1^ (Total Available/day) ^2^**	4560 ± 178	682 ± 27 (59%)	139 ± 11 (27%)	165 ± 8 (14%)
**LMC**	1125 ± 172	203 ± 23 (72%)	25 ± 3 (20%)	35 ± 1 (12%)
**Average Consumed ^3^/day**	2377 ± 1144	313 ± 152 (52%)	86 ± 48 (33%)	92 ± 42 (15%)
**DRI (g)/AMDR (%) ^4^(/day)**	2800–3200	338–488 (45–65%)	67–117 (20–35%)	75–262 (10–35%)

^1^ Individual meal packs or field rations consisted of a choice of a variety of foods and beverages from 18 menu items (6 breakfast, 6 lunch and 6 dinner/supper), which participants (n = 10) were provided for the 4-day duration of the field trial. ^2^ The total available/day corresponds to the total amount of nutrients available for consumption if the three ration packs (breakfast, lunch and dinner) were consumed in their entirety. ^3^ Consumed indicates the average amount of energy and macronutrients eaten during 24 h (as measured using the measured food intake/food waste collection method).^4^ Dietary Reference Intakes/Average Macronutrient Distribution Ranges [26] (AMDR) (for a moderately active to highly active 70 kg male); carbohydrates, fat and protein AMDR based on average 3000 kcal/day. The data were calculated using the energy contribution of 4 kcal/g for carbohydrate, protein and 9 kcal/g for fat. AMDR = Average Macronutrient Distribution Range; LMC = light meal combat.

**Table 3:** Energy and nutrient intakes from field ration consumed and discarded during the arctic-like field training exercise (n = 10).

Nutrient	Intakes from Field Rations	Discarded from Field Rations
**Energy (kcal/day)**	2377 ± 1144	2617 ± 781
**Carbohydrates (g/day)**	313 ± 152	431 ± 135
**Total Fat (g/day)**	86 ± 48	64 ± 20
**Saturated Fat (g/day)**	39 ± 14	22 ± 8
**Protein (g/day)**	92 ± 42	74 ± 26
**Fibre (g/day)**	22 ± 12	41 ± 16
**Total Sugar (g/day)**	174 ± 79	276 ± 91
**Vitamin A (μg/day)**	466 ± 187	343 ± 139
**Vitamin C (mg/day)**	106 ± 57	367 ± 153
**Calcium (mg/day)**	476 ± 227	580 ± 175
**Iron (mg/day)**	16 ± 7	25 ± 8
**Sodium (mg/day)**	3542 ± 1722	3263 ± 1105

Study participants (n = 10) were Class A Reservists, completing a CAF basic military qualification course during a 5-day winter weather (arctic-like) training exercise. Field dietary intake assessed for 4 days using measured food intake/food waste collection method. Intakes from field rations refer to consumption of foods and beverages from selected individual meal packs and light meal combat field rations. Discarded refers to unconsumed or partially consumed foods from rations. Data are presented as means ± standard deviation (SD).

**Table 4:** Individual total energy expenditure, energy intake, energy deficit and energy availability (n = 10).

Sex	Energy Expenditure (kcal/day)	Energy Intake (kcal/day)	Energy Deficit (kcal/day)	Energy Availability (kcal/kgFFM/day)
F	3911	1363	2548	82.7
M	5523	4690	833	95.1
F	5200	1212	3988	88.1
M	5230	1572	3657	93.0
M	3761	3266	495	58.8
M	5985	1454	4530	91.0
M	5090	2203	2886	68.3
M	5007	2914	2092	88.1
F	4429	3378	1050	84.6
F	5029	1716	3312	88.1
**Mean ± SD**	4917 ± 693	2377 ± 1144	2539 ± 1396	83.8 ± 11.5

Study participants (n = 10) were Class A Reservists, completing a CAF basic military qualification course during a 5-day winter weather (arctic-like) training exercise. Energy expenditure using doubly-labelled water was assessed in 10 participants. Field dietary intakes from rations assessed for 4 days using measured food intake/food waste collection method. Data are presented as means ± standard deviation (SD) for the 10 participants. kcal = kilocalories; kg = kilogram; FFM = fat free mass.

## Supplementary Materials

The following are available online at https://www.mdpi.com/2072-6643/12/6/1638/s1, Table S1: Percent of top five discarded food items, condiments and seasonings and beverages of selected field rations during the winter weather field trial.
