# Supplementary material for: Energy Balance of Canadian Armed Forces Personnel during an Arctic-Like Field Training Exercise
Source: Nutrients. 2020 Jun 2;12(6):1638. doi: 10.3390/nu12061638 (PMC7352380; doi:10.3390/nu12061638)
Supplement: Supplementary file 1 [file nutrients-12-01638-s001.pdf]

Version May 25th, 2020
Nutrients 794717 – Supplementary Data
Ahmed et al., Energy Balance of CAF in Cold Weather Condition

Supplementary Data on Discarded Rations

Rations Discarded

Participants discarded/stripped on average 2567 ± 678 kcal/day over the duration of the study. Vegetable rice, and raisin and spice oatmeal were most frequently discarded in comparison to other food items, while condiments and seasonings e.g. seasoning mixes, hot sauces and whiteners, made up a higher percentage of discarded items (**Supplementary Table 1**). Tea bags were discarded the most compared to other beverages (**Supplementary Table 1**). Top five ration items discarded by energy and macronutrient composition (total nutrient composition for all participants over 4 days) were as follows: drink crystals, bread/hamburger buns and peanut butter were the significant source of energy, carbohydrates, total fat and protein and beef jerky was one of the top sources of protein discarded. Vegetable rice, oatmeal, condiments/seasonings, and tea/coffee were the top ration items that were discarded most frequently. This study also identified bread and hamburger buns as significant sources of energy, carbohydrates, total fat and protein based on the collective contribution from amount available in the rations. Some of these items with a high frequency of being discarded are also available in large amounts within the ration pack. For example, bread/hamburger bun (not counting tortillas or corn bread) is available 14 times in a sample of 18 IMP menu items.

In contrast, there were a large number of ration items (e.g. tea bags, hot sauces) that were not significant sources of nutrients also being frequently discarded by participants, thus indicating the importance of evaluating the inclusion of these items within the ration pack. It is likely that the high frequency of discarded beverage items might be related to either the availability of water, the amount of which may have limited the reconstitution of these items or that the participants may not want to go to the bathroom, especially considering the weather conditions.

Supplementary Table 1. Percent of top five discarded food items, condiments and seasonings and beverages of selected field rations during the winter weather field trial.

**Main Entrées**	**% discarded of the total selected**
Vegetable Rice	100%
Raisin and Spice Oatmeal	100%
Mashed Potatoes (including onion)	95%
Bean Salad	87%
Apple and Brown Sugar Crunchy Cereal	86%
**Condiments and Seasonings**	
Seasoning Mix	100%
Whitener	97%
Sugar	94%
Frost Winter Cool Gum	93%
Hot Sauce	93%

**Beverages**	
Tea Bag	98%
Espresso Roast Coffee	93%
Instant Coffee	91%
Drink Crystals (combined varieties) †	82%
Nescafe Vanilla	71%

Study Participants (n=18) were Class A Reservists, completing a CAF basic military qualification course during a 5-day winter weather training exercise (average temperature -11°C, with a minimum of -22°C, a maximum of -2°C). Participants selected and consumed four days of rations (including individual meal packs and light meal combats). The table shows the percent of discarded food item, calculated as the amount of the food item discarded/stripped in relation to the frequency discarded. The discarded ration items are inclusive of items stripped prior to the training phase. † Drink Crystals (all varieties) includes the percentage of drink crystals (one package is counted as 21grams) that were discarded.
